# An Interesting Case of a Retrobulbar Cavernous Hemangioma

**Published:** 2016-12-22

**Authors:** Matthew A. Applebaum, Timothy E. Lee, Connor Barnes, Joshua B. Elston, David J. Smith

**Affiliations:** ^a^University of South Florida Morsani College of Medicine, Tampa; ^b^Division of Plastic Surgery, Department of Surgery, University of South Florida, Tampa

**Keywords:** cavernous hemangioma, cavernous venous malformation, orbital venous malformation, intraorbital mass, retrobulbar mass

## DESCRIPTION

A 41-year-old woman presented with an asymptomatic left infraorbital mass that had continuously grown over a 4-year period. A magnetic resonance image showed a retroseptal mass without involvement of the neurovasculature or intrinsic musculature of the eye. Excisional biopsy showed a well-circumscribed, benign vascular proliferation, consistent with a cavernous hemangioma or cavernous venous malformation.

## QUESTIONS

1. **What is a cavernous-hemangioma or cavernous venous malformation of the orbit?**
2. **How do orbital cavernous venous malformations present?**
3. **What are some differential diagnoses of orbital cavernous malformation?**
4. **What are the current diagnostic tools and treatment options for orbital cavernous malformations?**

## DISCUSSION

A cavernous venous malformation or cavernous hemangioma of the orbit represents approximately 6% of intraorbital/retrobulbar masses of the orbit. Comparatively, vasculogenic masses represent 17% of orbital masses.^1^ Typically, these malformations are classified as noninfiltrative and slow growing at a rate reported as 10% to 15% increase per year. Although the nomenclature is often confusing, the preferred term of these lesions is “cavernous venous malformations.”^2^ Histologically, dysplasia or hypercellularity is not seen and only show features of slow flow venous lesions that are typically lined by a single layer of endothelium and composed of large, thin vessels. They are differentiated from infantile hemangiomas histologically due to the adult form lacking in GLUT-1.^1,2^

Orbital cavernous venous malformations typically present in middle-aged individuals and will cause mass-effect symptoms such as proptosis, pain, diplopia, and visual disturbance by compression of the optic nerve. They may also be completely asymptomatic/found incidentally on imaging.^1,3,4^ In our patient, these venous malformations presented asymptomatically and caused only a deformity of the extraocular tissues, bringing it to the patient's attention.

Imaging helps establish a differential diagnosis of numerous benign and malignant masses. The differential diagnosis for orbital cavernous malformations is broad and includes various types of cysts, other vasculogenic lesions, peripheral nerve lesions, optic nerve and meningeal tumors, osseous versus lipocytic lesions, etc. Vasculogenic lesions with flow such as a carotid or dural cavernous fistulas, capillary hemangioma, or lymphangioma must be evaluated by imaging to prevent complication and to plan the surgical approach.^1^

**Figure 1:**
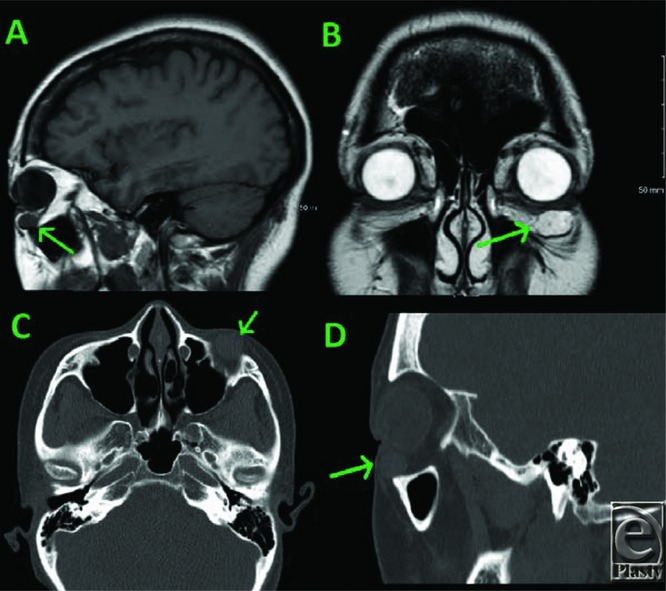
(a, b) T2- and T1-weighted magnetic resonance images. (c, d) Computed tomographic w/o contrast images of the orbital mass. Green arrow points to the orbital mass.

Obtaining a differential diagnosis is best achieved through computed tomography (CT), especially with the use of contrast dye that allows for the enhancement of the hemangioma. However, it is common for patients to sustain both magnetic resonance imaging (MRI) and CT as our patient did^2,5^ (Fig 1). Color Doppler and angiography may also help in the identification.^5,6^ A definitive diagnosis of orbital cavernous venous malformations requires surgical excision with confirmatory pathological analysis. However, the utilization of MRI and CT can help narrow the differential diagnosis.

**Figure 2:**
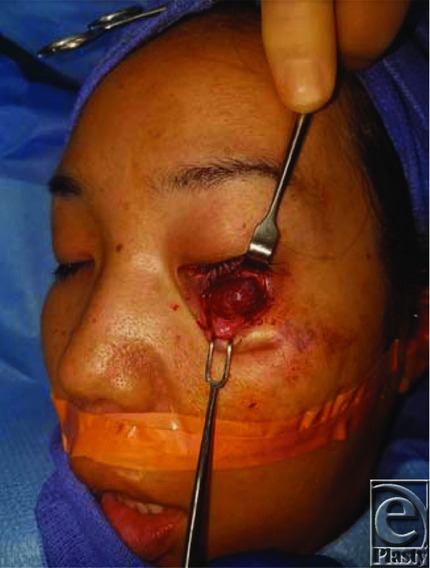
Subciliary approach to the left orbital mass.

**Figure 3:**
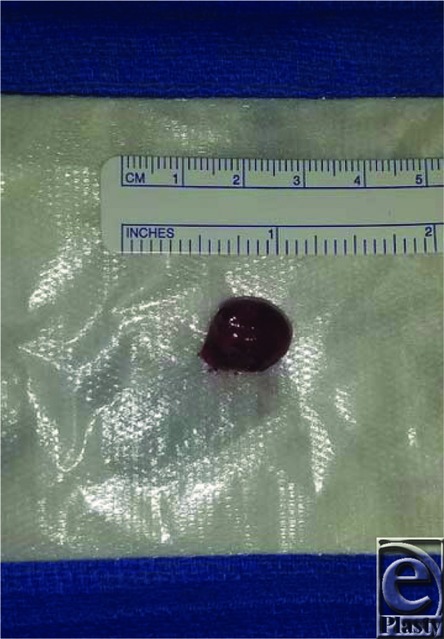
Gross examination of the left orbital cavernous venous malformation.

Treatment of orbital cavernous venous malformations can be either a nonsurgical method or various surgical excision methods. Location of the lesion also determines the recommended surgical approach. Nonsurgical methods may be indicated for small asymptomatic nonenlarging masses; however, surgical excision is required for definitive diagnosis. Lateral orbitotomy, supraorbital, transconjunctival, transantral, pterional, endoscopic, and extradural approaches have all been described as surgical approaches^6,7^ (Fig 2). The rate of recurrence is rare after surgical excision but has been reported in the literature.^1,3,5^ Gross examination of the patient's mass showed a tan-red, well-circumscribed mass in a fibrous membrane and measured 1.5 × 1.4 × 0.4 cm (Fig 3).
